# 116. Characterization of small colony variants from a patient with bloodstream infection of Candida glabrata

**DOI:** 10.1093/ofid/ofab466.116

**Published:** 2021-12-04

**Authors:** Shaoji Cheng, Badrane Hassan, Guojun Liu, Cornelius J Clancy, Minh-Hong Nguyen

**Affiliations:** University of Pittsburgh, Pittsburgh, PA

## Abstract

**Background:**

Bacterial small colony variants (SCVs) that are tolerant to commonly used antibiotics are well recognized. Clinical SCV *Candida* have been rarely reported. We describe SCV *C. glabrata* (CG) strains recovered from within a population causing bloodstream infection (BSI) in a patient (pt), which were not recognized by the micro lab. Pt J developed CG BSI shortly after liver transplant (OLTX), which was treated with voriconazole (VOR). VOR was also used for post-OLTX mold prophylaxis. 67 d after BSI, he developed intra-abdominal infection due to VOR-resistant CG. We hypothesized that BSI might be caused by an unrecognized mixed population of azole-susceptible and –resistant strains.

**Methods:**

Ten colonies from small (SCV) and large colonies (LC) from blood culture (BC) agar plates underwent Illumina NextSeq WGS and phenotype testing.

**Results:**

BCs from pt J harbored a diverse population of genetically distinct CG strains, differing by unique SNPs and indels [Fig. 1]. Gene variants identified were enriched for biological processes involved in mitochondrial processes (2.5e-9), cell adhesion (3.3e-5), and respiration (3.5e-4). Unlike LC, SCVs were fluconazole (FLU) resistant (MIC: 128 µg/mL), and exhibited enhanced *CDR1* and *PDR1* expression (257 ± 11, 15 ± 4, respectively). Compared to LCs, SCVs grew slowly in YPD, did not grow on media containing glycerol as sole carbon source, and were less adherent to agar. SCVs stained poorly with rhodamine 123 by fluorescence flow cytometry and had fewer mitochrondria by transmission electron microscopy, consistent with WGS findings and respiratory deficiency. SCVs were less susceptible to macrophage (J774) phagocytosis, and they were significantly outgrown by other strains in competitive infections *in vitro* and during disseminated candidiasis in mice. LCs incubated with FLU *in vitro* yielded SCVs in concentration-dependent manner. Likewise, LCVs passed through spleens of mice following IV inoculation yielded SCVs in both presence and absence of FLU.

Venn diagram for 5 representative stranis of C.glabrata

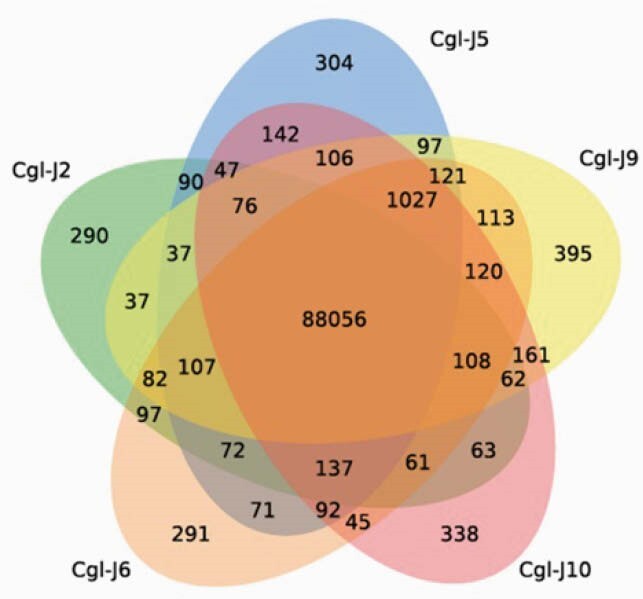

**Conclusion:**

Mitochondrial dysfunction and SCVs may be under-recognized determinants of azole resistance in *CG*, if micro labs select single colonies from BCs for antifungal susceptibility testing, or in absence of prolonged incubation.

**Disclosures:**

**Cornelius J. Clancy, MD**, **Merck** (Grant/Research Support) **Minh-Hong Nguyen, MD**, **Merck** (Grant/Research Support)

